# Endoscopic and pathologic motifs for the clinical diagnosis of Epstein–Barr virus‐associated gastric cancer

**DOI:** 10.1002/deo2.7

**Published:** 2021-04-21

**Authors:** Hideo Yanai, Daisuke Chihara, Megumi Harano, Eiki Sakaguchi, Tomoyuki Murakami, Jun Nishikawa

**Affiliations:** ^1^ Department of Clinical Research National Hospital Organization Kanmon Medical Center Yamaguchi Japan; ^2^ Department of Gastroenterology & Hepatology National Hospital Organization Kanmon Medical Center Yamaguchi Japan; ^3^ Department of Pathology National Hospital Organization Kanmon Medical Center Yamaguchi Japan; ^4^ Department of Laboratory Science Graduate School of Medicine, Yamaguchi University Yamaguchi Japan

**Keywords:** carcinoma with lymphoid stroma, Epstein–Barr virus, gastric cancer

## Abstract

**Objectives:**

Based on the recent therapeutic trends for gastric cancer (GC), the clinical impact of the diagnosis of Epstein–Barr virus (EBV)‐associated GC (EBVaGC) appears to be important. We retrospectively analyzed endoscopic and pathologic motifs of GC lesions to narrow the number of candidates for EBV testing.

**Methods:**

We performed EBV tests for 32 upper gastrointestinal lesions of 32 patients in the clinical setting. These tests were ordered by endoscopists or by pathologists without an endoscopist's order. EBV‐encoded small RNA1 (EBER1) in situ hybridization was used for the EBV tests. The endoscopic motif for the EBV test was the location in the upper part of the stomach or remnant stomach, mainly the depressed type with a submucosal tumor‐like protrusion of the lesion. The pathologic motif was carcinoma with lymphoid stroma (CLS) or CLS‐like histology of the lesion. We retrospectively analyzed the results of EBV tests for the endoscopic and pathologic motifs.

**Results:**

The final pathological diagnoses of the 32 subjects were 26 GCs including CLS, gastric endocrine cell carcinoma, gastric hepatoid carcinoma, gastric T‐cell lymphoma, gastritis of the remnant stomach, esophageal adenocarcinoma, and esophageal squamous cell carcinoma. When nontypical lesions were excluded, the EBER1‐positive rate was 42.3% (11/26) in GCs. Of the 14 GC lesions ordered examined by endoscopists, three (21.4%) were EBVaGC. Eight of the 12 (66.7%) GCs ordered examined by pathologists were EBVaGC.

**Conclusions:**

The pathologic motif is expected to be useful and the endoscopic motif may be helpful for EBVaGC diagnosis.

**Abbreviations**
CLS: carcinoma with lymphoid stroma
EBV: Epstein–Barr virus
ESD: endoscopic submucosal dissection
GC: gastric cancer

## INTRODUCTION

Recently, monoclonal Epstein–Barr virus (EBV) infection of all cancer cells is detected in almost 10% of gastric cancer (GC) lesions and a causal association of EBV with GC is suspected. In EBV‐associated GC (EBVaGC) lesions, EBV‐encoded small RNA1 (EBER1) is detected in all cancer cell nuclei by using EBER1 in situ hybridization (EBER1 ISH). EBVaGC characteristically shows extreme DNA hypermethylation and overexpression of programmed cell death‐ligand 1 and ligand 2. Histologically, the EBVaGC lesion is mainly poorly differentiated, tumor‐infiltrating lymphocyte‐rich adenocarcinoma (carcinoma with lymphoid stroma [CLS]). Many EBVaGC lesions are located in the upper part of the stomach and remnant stomach. Endoscopically, some EBVaGC lesions have a submucosal tumor (SMT)‐like protrusion consisting of a CLS mass.^1^, ^2^, ^3^, ^4^, ^5^

The treatment outcome of advanced EBVaGC is reported to be better than that of EBV‐negative GC and immune‐checkpoint inhibitor therapy is promising.^6^, ^7^, ^8^

Currently, less‐invasive endoscopic submucosal dissection (ESD) is widely used for early GC worldwide. Accumulating data show that the risk of lymph node metastasis from early EBVaGC is low. Thus, further extension of the criteria of ESD for early EBVaGC should be discussed.^9^, ^10^, ^11^, ^12^, ^13^ In many cases, EBVaGC is located in the upper part of the stomach or remnant stomach. Therefore, such extension of the ESD criteria may prevent possible excessive surgery such as total gastrectomy.

Based on the recent therapeutic trends for GC, the clinical impact of EBV detection in GC lesions appears to be important. In the clinical setting, however, EBV testing for all GC lesions is not practical. Therefore, we retrospectively analyzed endoscopic and pathologic motifs in GC lesions in an attempt to determine criteria to narrow the number of candidates for EBV testing.

## METHODS

We performed EBV tests for 32 gastric or esophagogastric lesions of 32 patients at the National Hospital Organization Kanmon Medical Center from January 2008 to December 2017. These tests were ordered in the real‐time clinical setting by endoscopists or by pathologists without an endoscopist's order. EBER1 ISH was used for EBV testing of endoscopic biopsy specimens and/or surgically resected specimens. The endoscopic motif for the EBV test was the location in the upper part of the stomach or remnant stomach, mainly the depressed type with SMT‐like protrusions of the lesions. The pathologic motif was CLS or CLS‐like histology of the lesions.

We retrospectively analyzed the results of EBV tests done based on the endoscopic or pathologic motif.

We obtained written informed consent from all 32 patients in accord with the principles of the Declaration of Helsinki and the study was approved by the institutional ethical committee (H2801‐2).

## RESULTS

**FIGURE 1:**
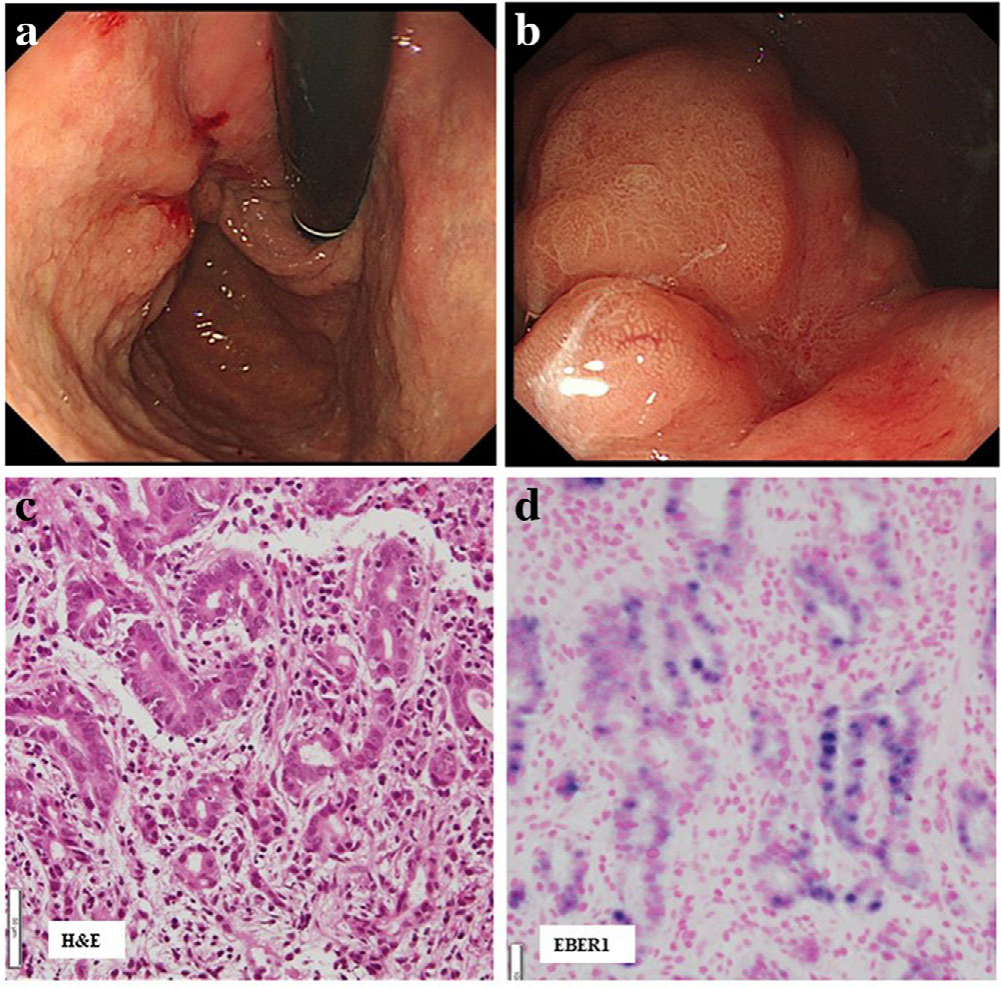
Inoperable advanced EBVaGC case, 71‐year‐old male. The endoscopist ordered an EBV test of the endoscopic biopsy specimen because of the endoscopic motif. His GC lesion was EBER1 positive. His disease was inoperable because of multiple distant metastases and he underwent chemotherapy. There was no surgical specimen and the main histologic type of his GC lesion is unknown. (a, b) Endoscopic features. There was mainly a depressed‐type GC lesion surrounded by an SMT‐like protrusion in the upper part of the stomach. The endoscopist ordered an EBV test because of the endoscopic motif. (c) Pathologic features of endoscopic biopsy specimen, H&E stain. The pathological diagnosis was moderately differentiated adenocarcinoma of the stomach. CLS was not observed. (d) EBER1 result. Nuclei of carcinoma cells were EBER1 positive and the GC lesion was diagnosed as EBVaGC

The patients consisted of 24 males and eight females. Their mean age was 72.9 years and ranged from 56 to 88 years. The final pathological diagnoses of the 32 subjects were 26 GCs including CLS, gastric endocrine cell carcinoma, gastric hepatoid carcinoma, gastric T‐cell lymphoma, gastritis of the remnant stomach, esophageal adenocarcinoma, and esophageal squamous cell carcinoma. When such nontypical gastric tumors and esophageal cancers were excluded, the EBER1‐positive rate was 42.3% (11/26) (Table 1). Endoscopists ordered EBV tests for 18 lesions with the endoscopic motif. EBV was negative for four lesions of non‐GC (gastric endocrine cell carcinoma, gastric hepatoid carcinoma, gastric T‐cell lymphoma, and gastritis of the remnant stomach). Of the remaining 14 lesions of GC, three (21.4%) were EBVaGC. One of these three EBVaGC was inoperable advanced GC, so only an endoscopic biopsy specimen was available for the EBV test. The biopsy specimen did not exhibit CLS, but an EBV test was ordered based on the endoscopic motif and the lesion was identified as an EBVaGC (Figure 1).

**TABLE 1:** The final pathological diagnoses and the results of the EBV test for the 32 lesions

**Pathological diagnosis**	**EBV test**	
Gastric carcinomas including carcinoma		
with lymphoid stroma (CLS),	26 lesions	42.3% (11/26) were EBV positive
tested with endoscopic motif,	14 lesions	21.4% (3/14) were EBV positive
pathologic motif	12 lesions	66.7% (8/12) were EBV positive
Gastric endocrine cell carcinoma, 1 lesion		
endoscopic motif		Negative
Gastric hepatoid carcinoma, 1 lesion		
endoscopic motif		Negative
Gastric T‐cell lymphoma, 1 lesion		
endoscopic motif		Negative
Gastritis of the remnant stomach, 1 lesion		
endoscopic motif		Negative
Esophageal adenocarcinoma, 1 lesion		
pathologic motif		Negative
Esophageal squamous cell carcinoma, 1 lesion		
pathologic motif		Negative

EBV was negative in 11 of the GC lesions tested based on the endoscopic motif. In these GC lesions with false‐positive endoscopic motifs, there were two GCs of the remnant stomach, eight GCs of the depressed type located in the upper part of the stomach, and one GC located in the lower part of the stomach. The GC lesions with false‐positive endoscopic motifs included five GCs with an SMT‐like protruding part. The pathologic features of these five endoscopic false‐positive SMT‐like protrusions were mucinous adenocarcinoma, submucosal cyst, fibrosis, CLS‐like lymphocytic infiltration, and lymphoma‐like lymphocytic infiltration.

**FIGURE 2:**
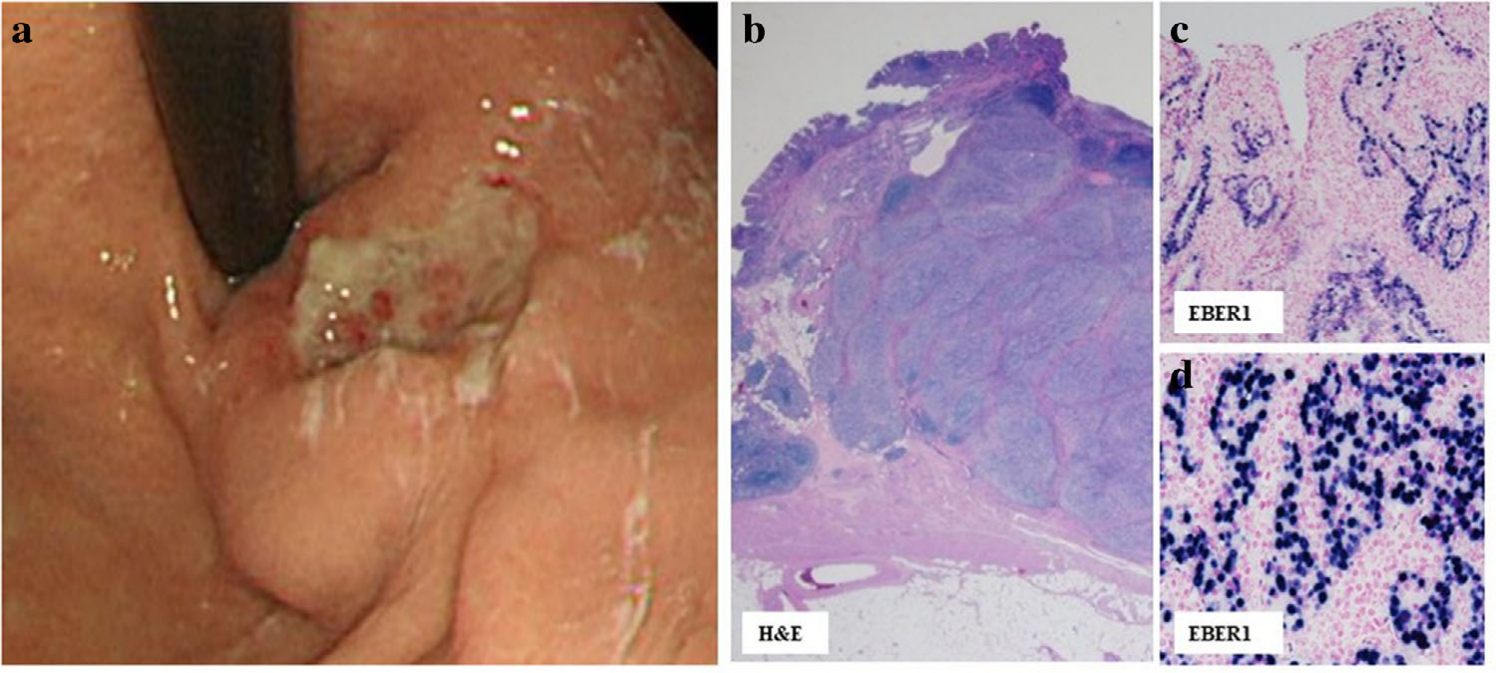
Surgically resected advanced EBVaGC case, 71‐year‐old female. A pathologist ordered an EBV test because of the pathologic motif. Her GC lesion was EBVaGC. (a) Endoscopic features. The GC lesion was mainly the depressed type with an SMT‐like protrusion located near the gastric cardia. The endoscopist did not order an EBV test because of the lack of sufficient knowledge of EBVaGC. This GC lesion had the endoscopic motif for the EBV test on retrospective review. (b) Low‐power view of pathologic features with H&E stain. CLS had SMT‐like thickening of the gastric wall. There was also differentiation on the tumor surface. A pathologist ordered an EBV test because of the CLS pathologic motif. (c, d) Tumor cell nuclei were EBER1 positive in both the CLS portion (d) and differentiated adenocarcinoma portion (c)

There were 14 lesions for which pathologists ordered EBV tests without an endoscopist's request. These consisted of three endoscopic biopsy specimens and 11 surgically resected specimens. Two non‐GC lesions of esophageal adenocarcinoma at the esophagogastric junction and esophageal squamous cell carcinoma were EBV negative. Eight (66.7%) of the other 12 GCs were EBVaGC. Of eight CLS GC lesions, six (75%) were EBVaGC. Of four CLS‐like GC lesions, two (50%) were EBVaGC. Five of the eight EBVaGCs correctly detected based on the pathologic motif were found to have the endoscopic motif of EBVaGC retrospectively (Figure 2).

## DISCUSSION

In the present study, four lesions with false‐positive endoscopic motifs (endocrine cell carcinoma, hepatoid carcinoma, T‐cell lymphoma, and gastritis of the remnant stomach) were not ordinary GCs. In these lesions, gastric wall thickening with tumors or inflammation was responsible for the false‐positive endoscopic motifs. Thus, these nontypical gastric tumors and such inflammation may be important in the differential diagnosis of EBVaGC. Two lesions ordered tested by pathologists without endoscopists orders (CLS‐like esophageal adenocarcinoma and esophageal squamous cell carcinoma) were EBV negative. Reportedly, esophageal adenocarcinoma and esophageal squamous cell carcinoma are EBV‐negative lesions. Therefore, in the future, such esophageal carcinomas at the esophagogastric junction should be excluded from the motif for EBV testing.^17^, ^18^ When such atypical gastric tumors and esophageal cancers were excluded, the EBER1‐positive rate was 42.3% in GCs.

EBV testing may be especially needed in inoperable advanced GC cases or early GC cases not included in the present curative ESD indication.^7^, ^13^

In a meta‐analysis, Murphy et al. reported that the rates of EBVaGC were 13.6% in the gastric cardia and 13.6% in the gastric body.^2^ Our EBVaGC rate of 21.4% with the endoscopic motif was favorable, but not sufficiently high. In our 11 endoscopic false‐positive cases, the histologic backgrounds were mucinous adenocarcinoma, submucosal glands, fibrosis, and lymphoid infiltration. For such endoscopic false‐positive lesions, endoscopic ultrasonography may be useful.^13^, ^19^ It is clear that further progress in endoscopic diagnosis of EBVaGC is needed.

The EBVaGC rate of 66.7% for EBV detection in lesions with the pathologic motif was thought to be sufficiently high. In CLS lesions, especially, the EBVaGC rate was 75% and the pathologic motif of CLS is expected to be useful. The treatment outcome of advanced EBVaGC is reported to be better than that of EBV‐negative GC.^6^ Therefore, an EBV test for surgically resected specimens may be useful before additional treatment such as adjuvant chemotherapy.

However, some CLS lesions have an ordinary differentiated adenocarcinoma component on the surface of the lesion.^20^ In such lesions, biopsy specimens may lack the histologic features of CLS and this may result in a false‐negative for the pathologic motif. Actually, in the present study, one inoperable advanced GC lesion and two GC lesions of the remnant stomach had non‐CLS differentiated‐type histology in biopsies and were diagnosed as EBVaGC with the endoscopic motif. We have already reported a case with a favorable outcome using chemotherapy against inoperable advanced EBVaGC. Furthermore, inoperable advanced EBVaGC may be a potential candidate for immune‐checkpoint inhibitor therapy.^7^, ^8^ In such inoperable GC cases without surgical specimens, the endoscopic motif may be an important indication for EBV testing. Five of eight EBVaGC lesions with the pathologic motif for which EBV testing was conducted were found to also have the endoscopic motif in a retrospective review of the endoscopic features. This suggests that knowledge of the endoscopic motif of EBVaGC is important for endoscopists. Combination of the endoscopic and pathologic motifs seems to be useful for the diagnosis of EBVaGC.

The present study has limitations since it was a retrospective analysis with a small sample size and subjective biases. However, the pathologic motif is expected to be useful and the endoscopic motif may be helpful as indicators for EBV testing in the diagnosis of EBVaGC. A future prospective study using a visual analog scale for the endoscopic and pathologic motifs or application of artificial intelligence for the diagnosis of EBVaGC may be needed to verify our conclusion.

## CONFLICT OF INTEREST

The authors declare that there is no conflict of interest.

## FUNDING INFORMATION

None.
